# Elemental Design of Alkali-Activated Materials with Solid Wastes Using Machine Learning

**DOI:** 10.3390/ma17184573

**Published:** 2024-09-18

**Authors:** Junfei Zhang, Shenyan Shang, Zehui Huo, Junlin Chen, Yuhang Wang

**Affiliations:** 1 School of Civil and Transportation Engineering, Hebei University of Technology, Tianjin 300401, China; 2 Arizona College of Technology, Hebei University of Technology, Tianjin 300401, China; junlinchen@hebut.edu.cn; 3 Institute of Information Engineering, Chinese Academy of Sciences, Beijing 100085, China

**Keywords:** fly ash, granulated blast furnace slag, alkali-activated materials, strength, machine learning

## Abstract

Understanding the strength development of alkali-activated materials (AAMs) with fly ash (FA) and granulated blast furnace slag (GBFS) is crucial for designing high-performance AAMs. This study investigates the strength development mechanism of AAMs using machine learning. A total of 616 uniaxial compressive strength (UCS) data points from FA-GBFS-based AAM mixtures were collected from published literature to train four tree-based machine learning models. Among these models, Gradient Boosting Regression (GBR) demonstrated the highest prediction accuracy, with a correlation coefficient (R-value) of 0.970 and a root mean square error (RMSE) of 4.110 MPa on the test dataset. The SHapley Additive exPlanations (SHAP) analysis revealed that water content is the most influential variable in strength development, followed by curing periods. The study recommends a calcium-to-silicon ratio of around 1.3, a sodium-to-aluminum ratio slightly below 1, and a silicon-to-aluminum ratio slightly above 3 for optimal AAM performance. The proposed design model was validated through laboratory experiments with FA-GBFS-based AAM mixtures, confirming the model’s reliability. This research provides novel insights into the strength development mechanism of AAMs and offers a practical guide for elemental design, potentially leading to more sustainable construction materials.

## 1. Introduction

The carbon dioxide (CO_2_) emissions generated by the construction industry constitute 38% of the total emissions related to energy production globally, with nearly half of this proportion attributed to cement production [1,2,3]. These carbon emissions are part of greenhouse gases, causing adverse effects on global climate change [4,5]. Additionally, the cement production process is accompanied by the emission of other air pollutants such as nitrogen oxides and sulfur oxides, which may have detrimental impacts on air quality and ecosystem health. Consequently, there is an imperative to identify a novel material to substitute ordinary portland cement (OPC) [6]. In contrast to OPC, alkali-activated materials (AAMs) not only offer superior performance but also effectively address the issue of excessive CO_2_ emissions associated with OPC production [7].

AAMs, fundamentally, are an inorganic polymer formed through the alkaline activation of aluminosilicate materials, undergoing a series of activation reactions [8]. In terms of raw materials, industrial by-products such as granulated blast furnace slag (GBFS), fly ash (FA), metakaolin, and the slurry from ceramic tile production offer significant potential as precursors for geopolymers. This slurry contains fine fractions of kaolinite and quartz, which are highly reactive under alkaline conditions [9]. Simultaneously, alkaline activators such as sodium hydroxide, water glass, and sodium carbonate, chosen for their cost-effectiveness, are widely employed [8,10,11,12,13]. AAMs not only exhibit excellent mechanical properties, including compressive, tensile, and shear strengths, but also display high resistance to chemical corrosion [14,15,16,17,18,19]. As described above, the ability to reuse industrial waste products like GBFS and FA, coupled with the use of cost-effective alkaline activators, allows for the recycling of waste solids. It is precisely these characteristics of AAMs that drive their widespread application and gradual substitution for OPC, offering significant commercial and societal benefits [20,21,22,23]. In addition, AAMs are quite sensitive to environmental temperature and humidity changes and require high precision during mixing and construction. The long-term stability of AAMs needs to be further studied [24].

To address the problems related to mechanical properties and durability, mixture optimization methods should be developed. In terms of precursors, the elemental composition ratios within various materials, such as Ca/Si, Na/Al, Si/Al ratios, and water content (WC), play a predominant role in the development of UCS of AAMs [25]. Regarding environmental conditions, the uniaxial compressive strength (UCS) development of AAMs is primarily influenced by curing periods (CP), curing temperature (CT), and humidity (H) [26]. In the process of strength formation in AAMs, the hydration reaction is a crucial step. This involves the reaction of minerals in the materials with water, leading to the formation of a gel that primarily determines its strength through its formation and structure. Additionally, the interaction between water and the gel is another important influencing factor [27].

The challenge lies in the fact that, whether in terms of precursor-related factors or environmental factors, the impacts generated by these factors on the development of UCS in AAMs are not mutually independent but rather intricately intertwined and complex [25]. This has also led to the realization that traditional trial-and-error methods in the laboratory not only consume significant human and financial resources but also fall far short of meeting the time demands. The limitations of traditional laboratory trial-and-error methods have been glaringly exposed [28,29,30,31]. Some have attempted to substitute laboratory trial-and-error methods with formulaic analytical approaches and statistical methods, but the ultimate results have not proven to be very satisfactory [32]. The former is based on a clear relationship between the relevant empirical formulas and the components of the system, both computationally and analytically, and lacks flexibility in practical application [33,34], while the latter requires a large amount of laboratory mix data to fit the model, but even so, there are still shortcomings in predictive performance and other functions [35,36].

In order to solve this problem, advanced machine learning (ML) methods can be used to assist in the mixture design of AAMs [25]. ML algorithms such as support vector machine (SVM), random forest (RF), and artificial neural network (ANN) can be used to model without knowing the explicit relationship and accurately predict the UCS, dry shrinkage, and air permeability of concrete [37,38,39,40]. The method of ML greatly compensates for the shortcomings of the previously mentioned methods and opens up new possibilities for the development of the field of civil engineering.

Nguyen et al. [31] employed a deep neural network to forecast the compressive strength of FA-based AAMs and obtained good prediction accuracy. Tanyildizi et al. [41] successfully used deep long short-term memory (LSTM) to forecast the dissolution peak heat, dissolution peak time, polymerization peak heat, and polymerization peak time of FA-based AAMs. Zhang et al. [42] proposed a chemical engineering feature based on the working performance of ML models such as gradient enhancement and extra trees, and the results showed that the prediction performance was very accurate. Huo et al. [43] constructed a tree-like ensemble model based on multiple regression models and successfully realized the multi-objective optimization of fly ash-slag base polymers combined with the non-dominant ranking genetic algorithm. However, the above studies only predicted the strength of alkali-activated materials from a macroscopic perspective (such as precursor content and curing periods, etc.) or optimized the characteristics of AAMs but did not explain the strength formation mechanism of AAMs from a microscopic perspective. The ML models can interpret the influence of composition variables through various techniques and approaches, such as the SHapley Additive exPlanations (SHAP) [44]. SHAP values provide a way to interpret the impact of each feature on an individual prediction. These methods are grounded in cooperative game theory and offer a cohesive metric for evaluating feature significance. Therefore, this paper uses ML techniques to reveal the strength development mechanism and facilitate the elemental design of AAMs.

This research bridges significant knowledge gaps by providing a clearer understanding of the microscopic mechanisms underlying AAM strength development and by offering practical solutions to optimize the design and performance of AAMs. Additionally, the study addresses the environmental and economic challenges associated with AAMs, contributing to the advancement of sustainable construction materials [45]. We use the Ca/Si ratio, Na/Al ratio, Si/Al ratio, water content, curing periods, curing temperature, and humidity as input variables to train ML models from the datasets compiled in the published literature, optimize their hyper-parameters through the Bayesian optimization algorithm (BOA), comprehensively analyze the final results, and put forward the findings of this study, hoping to promote the discussion of scholars in AAM and other fields.

## 2. Dataset Description

In this paper, based on a large number of previous studies on AAMs [46,47,48,49,50,51,52,53,54,55,56,57,58,59,60,61,62,63,64,65,66,67,68,69], 616 data instances with water content, Ca/Si ratio, Na/Al ratio, and Si/Al ratio were selected as internal factors, and curing periods, curing temperature, and humidity were selected as external factors [26]. The data are included in the Supplementary Materials in this paper in this paper. The following are the criteria used in this article to select datasets:

(1) The UCS data were extracted from studies published in internationally recognized journals, ensuring that the data used in this research were of high quality and peer-reviewed.
(2) Only datasets involving AAMs using FA and GBFS as precursors were considered. This selection criterion was established to maintain consistency in the chemical composition and reaction mechanisms across all samples, thereby eliminating the influence of other precursor materials such as metakaolin or rice husk ash.
(3) The dataset excluded any AAMs that included aggregates in their composition. This decision was made to focus solely on the binder’s properties without the additional variability introduced by different aggregate types.
(4) All selected data pertained to AAMs that were formulated and cured under controlled laboratory conditions. Specifically, the samples were prepared and cured in curing chambers, ensuring uniform curing periods, temperatures, and humidity levels. This consistency is crucial for the accurate modeling of UCS development.
(5) Only studies that clearly reported the chemical composition and proportions of the alkali activators used (e.g., sodium hydroxide, sodium silicate) were included. This transparency allows for precise replication of the mix design and activation process in future studies.

The data collection process involved meticulously extracting data points from each study, ensuring that all relevant variables, such as precursor ratios, water content, curing periods, and environmental conditions, were accurately recorded and then organized into a standardized format for analysis. Prior to analysis, the dataset underwent thorough processing to identify and correct any inconsistencies, including discrepancies in units of measurement or reporting formats, and outliers were carefully examined to determine whether they were experimental errors or legitimate variations in material behavior. The selected input variables for the machine learning models, including the Ca/Si ratio, Na/Al ratio, Si/Al ratio, water content, curing temperature, curing periods, and humidity, were chosen based on their recognized influence on the strength development of AAMs, as identified in the literature [70,71].

**Figure 1:**
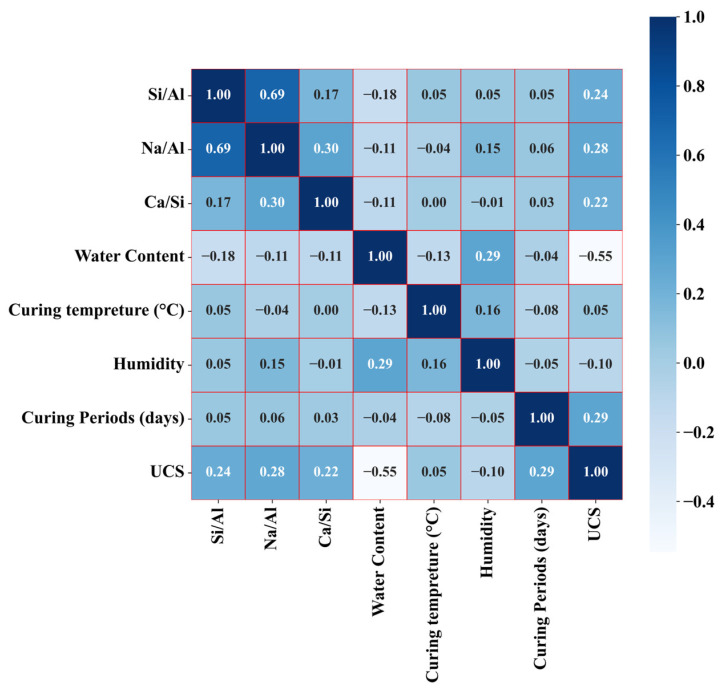
Correlation matrix of each input variable in the dataset.

The selected data are organized into a complete data set, and the input variables and UCS statistics are listed, as shown in Table 1. Figure 1 displays the correlation coefficient matrix among the input variables, revealing the degree of interdependence among them. Positive values denote a positive correlation, while negative values indicate the opposite, with larger absolute values signifying stronger correlations. As can be seen from Figure 1, the absolute value of the correlation coefficient between most of the input variables remains below 0.5, which indicates that the input variables maintain good independence as a whole.

## 3. Methodology

### 3.1. Machine Learning Methods

Tree-based ensemble models work by combining multiple tree-like models with each other to improve the prediction performance and stability of the overall model, which is superior to traditional empirical models, and such ensemble methods are widely used in classification, regression, and feature selection [72,73,74]. In this study, Random Forest Regression (RFR), Extremely Randomized Trees (ERT), Gradient Boosting Regression (GBR), and Extreme Gradient Boosting Regression (XGBR) are employed, and their performance on UCS prediction of AAMs is compared.

Tree-based ensemble models were chosen for this study due to their ability to significantly improve prediction performance and stability compared to traditional empirical models. These models combine multiple decision trees, each trained on different subsets of the data, to form an ensemble, which reduces variance and prevents overfitting—an issue often encountered with single decision trees [25]. This approach is particularly suited for handling the complex and non-linear interactions between variables in AAMs, such as precursor ratios, curing conditions, and environmental factors. Unlike traditional empirical models, which rely on predefined equations that may not fully capture these interactions, ensemble models can learn these relationships directly from the data without explicit assumptions. Moreover, ensemble methods like Random Forest and Gradient Boosting offer valuable insights into feature importance, helping to identify which variables most significantly impact strength development in AAMs. This adaptability to different data types and the ability to generalize better to unseen data make tree-based ensemble models a powerful and flexible tool for modeling the intricate relationships in AAMs, ensuring more accurate and reliable predictions of UCS.

#### 3.1.1. Random Forest Regression

Random Forest Regression (RFR) is a common decision tree-based ensemble ML method [75,76]. By constructing a large number of decision trees during the training phase, decision tree ensembles (hereinafter referred to as ‘ensembles’) are formed. The features used in constructing each decision tree (input variables of various classes in the model) are all random (randomly sampled using the Bagging model). Here, we define T as the total dataset, in which there are N samples. The training set comprises d features, and only $k\left(k<d\right)$ is selected for building the decision tree at a time. The following is the procedure to establish a random forest regressor.

1. From the total dataset T, N samples are randomly selected for training a decision tree. As they are put back in the selection process, it is almost impossible to select all the samples, although N samples are selected, and each choice will not be exactly the same. These N samples serve as the data points at the root node of the decision tree.
2. At each node where a decision tree requires splitting, $m\left(m<M\right)$ attributes are randomly selected from the M attributes, given that each sample has M attributes. Subsequently, one attribute is chosen from this subset of m attributes to serve as the splitting attribute for that particular node.
3. Step (b) is repeated until the decision tree can no longer be divided, and the entire decision tree is not pruned during the formation process.
4. By repeating steps (a)~(b), an extensive array of decision trees is constructed to form random forests.
5. Randomly selecting features and a subset of data to train all decision trees in the set helps reduce the correlation between each tree in the set and prevents overfitting.
6. The average of all decision tree predictions in the set is taken as the final regression value of the model.

Random forests are robust to missing and outliers. In the face of missing and outliers, they can process datasets that mix numeric and categorical features and calculate the relative importance of the features in question.

#### 3.1.2. Extremely Randomized Trees

Extremely Randomized Trees (ERT), also known as extra tree, is a new extension algorithm for RFR [77,78]. ERT also uses the Bagging model; however, the distinction lies in the fact that ERT utilizes all the training samples to construct each decision tree, meaning each decision tree applies the entire set of training examples uniformly. RFR selects the best features and their corresponding values in a random subset to obtain the best bifurcation attributes. In contrast, ERT achieves the branching of the decision tree by randomly selecting the split values, ensuring a random bifurcation process. Compared with RFR, this bifurcation method emphasizes randomness, reduces variance, and improves the robustness of the model.

#### 3.1.3. Gradient Boosting Regression

Gradient Boosting Regression (GBR) is an ensemble learning method based on the Boosting model, also known as the Gradient Boosting Decision Tree algorithm. It is characterized by high predictive accuracy and stability [79]. To build GBR, a weak learner (typically a decision tree) is built on the original dataset. Then, this weak learner is used for predictions, and then the differences between the actual values and the predicted values (residuals) are computed. Subsequently, the next weak learner continues to be trained, and the residuals of the previous weak learner are predicted. The above process is repeated until training a predetermined quantity of weak learners or achieving a certain level of predictive performance. The ultimate prediction is derived by summing up the predictions generated by all weak learners. The learning rate parameter can control the step size during model optimization, and theoretically, a smaller learning rate can improve the predictive performance of the algorithm but may also increase the number of iterations during model optimization.

#### 3.1.4. Extreme Gradient Boosting Regression

Extreme Gradient Boosting Regression (XGBR) is similar to GBR in that it is essentially k classification and regression trees (CART), where k is a positive integer [80]. The loss function in XGBR employs a second-order Taylor expansion, which is more accurate than GBR (first-order Taylor expansion).

Overfitting is prevented through L1 regularization (penalizing weights based on the sum of their absolute values) and L2 regularization (penalizing weights based on the sum of squares of weights). This process continuously builds new weak learners to fit and reduces the residuals of the previous weak learners until the specified criteria are met. The predicted values from all weak learners are averaged as the final output of the algorithm.

### 3.2. Hyper-Parameter Tuning

The hyper-parameter tuning algorithm used in this article is the BOA. BOA distinguishes itself from Grid search and Random search by leveraging information from previously explored points to inform the selection of the next search point. This adaptive approach enhances both the quality and efficiency of the search process by intelligently guiding it based on past observations [81,82]:

(1) $$p\left(wD\right)=\frac{p\left(Dw\right)p\left(w\right)}{p\left(D\right)}$$

where $p\left(w\right)$ and $p\left(wD\right)$ denote the prior and posterior distributions, respectively; $p\left(Dw\right)$ represents the probability, and w is the unseen data.

**Figure 2:**
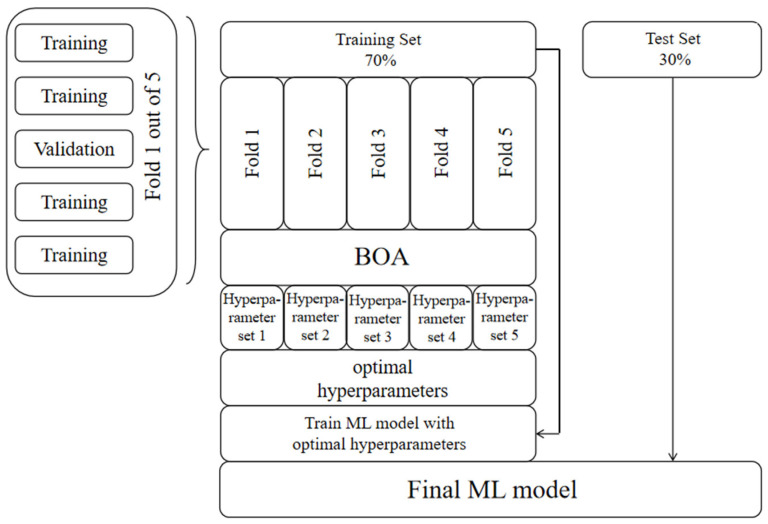
Hyper-parameter tuning procedure.

In detail, the entire dataset is partitioned in a 7:3 ratio, allocating 70% of the instances to the training set and 30% to the test set. Five-fold cross-validation is used to avoid overfitting [83]. The training set undergoes division into five subsets. In each fold, the BOA seeks the optimal hyper-parameters of the machine learning algorithm within four of these subsets and calculates the mean absolute error (MAE) on the validation set (the last subset) to describe the model performance under this hyper-parameter. The above process is repeated five times, and the validation set is different each time, and finally the average of the five sets of hyper-parameters is used as the final hyper-parameter (Figure 2).

Note that, for tree-based ML models, the number of weak learners (n_estimators) can significantly impact the performance of the ML model. In particular, for GBR and XGBR models, the learning rate is a critical factor influencing the performance of the machine learning model. Therefore, it is essential to carefully balance these two hyper-parameters, learning rate and the number of weak learners, to achieve optimal robustness for the machine learning model. The range of hyper-parameter tuning for each model is shown in Table 2.

### 3.3. Performance Evaluation Methods

For the convenience of providing an intuitive assessment of the predictive performance of various ML models, this paper employs the correlation coefficient (R), coefficient of determination (R^2^), mean absolute error (MAE), and root mean square error (RMSE) as evaluation metrics. The following are their definitions:

R is the product of the covariance/standard deviation of the independent variable X and the dependent variable Y and is a measure of the linear correlation between the variables [84].

(2) $$R=\frac{\sum_{i=1}^{n}\left({y_{i}}^{*}-{\bar{y}}^{*}\right)\left(y_{i}-\bar{y}\right)}{\sqrt{\sum_{i=1}^{n}{\left({y_{i}}^{*}-{\bar{y}}^{*}\right)}^{2}}\sqrt{\sum_{i=1}^{n}{\left(y_{i}-\bar{y}\right)}^{2}}}$$

where ${\bar{y}}^{*}$ and $\bar{y}$ are the means of the predicted and actual values, respectively; *n* is the number of instances.

$R^{2}$ represents the goodness of fit of regression model coefficients after performing linear regression on the model [84].

(3) $$R^{2}=\frac{SSR}{SST}=1-\frac{SSE}{SST}$$

where *SST* is the total sum of squares; *SSR* is the regression sum of squares; *SSE* is the error sum of squares.

MAE is the average value of the difference between the predicted value and the true value, which can better reflect the actual situation of the error of the predicted value [84].

(4) $$MAE=\frac{1}{m}\sum_{i=1}^{m}\left|Y_{i}-{\hat{Y}}_{i}\right|$$

where $Y_{i}$ and ${\hat{Y}}_{i}$ denote observed and predicted outputs; *m* is the number of instances.

RMSE represents the deviation between the estimated value and the target value, and unlike MAE, RMSE is the L2 specification and MAE is the L1 specification, which results in RMSE having a higher sensitivity than MAE. When there are many outliers in the dataset, RMSE will show a high value, which is often used as a measure of the prediction results of ML models [84]:

(5) $$RMSE=\sqrt{\frac{1}{n}\sum_{i=1}^{n}{\left({y_{i}}^{*}-y_{i}\right)}^{2}}$$

where $y_{i}$ and ${y_{i}}^{*}$ denote the actual and predicted values, respectively; *n* refers to the number of instances.

### 3.4. SHAP Analysis

ML models belong to a highly complex category of ‘black-box’ models, and the internal opacity of ML algorithms results in the difficulty in interpretability of the majority of ML models. In order to gain a deeper understanding of the working principles of ML models, this paper introduces Shapley Additive Explanation (SHAP) as a tool for explaining ML models. SHAP, a “model interpretation” tool, interprets the output of any machine learning model by treating all input features as contributors. For each prediction sample, the model generates a prediction value, with the SHAP value representing the contribution of each feature in that sample [85]. The SHAP values for changes in the model output due to variations in input features follow the following rules [86]:

(6) $$\varphi^{j}\left(f\right)=\sum_{S\subseteq \left\{x^{1},\ldots ,x^{p}\right\}\left\{x^{j}\right\}}\frac{\left|S\right|!\left(p-\left|S\right|-1\right)!}{p!}\left(f\left(S\cup \left\{x^{j}\right\}\right)\right)-f\left(S\right)$$

where $x^{j}$ is the feature variable; p is the number of features. *S* denotes a subset of the features and $f\left(x^{j}\right)$ is the output of the model.

**Figure 3:**
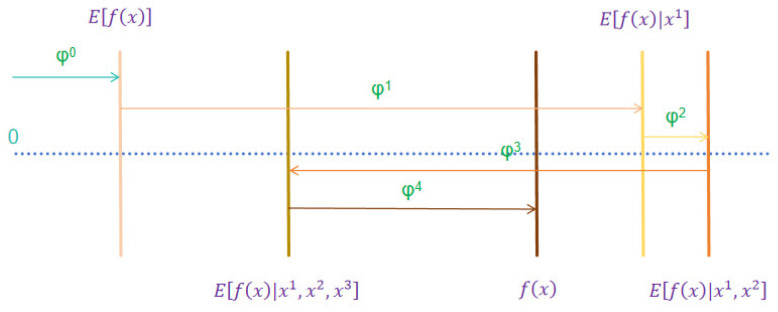
SHAP attributes.

The SHAP importance coefficient for a specific input feature is calculated by altering the input value of that feature and measuring the resulting prediction error. SHAP treats the output model as an interpretable model by linearly adding input variables and uses an additive feature attribution method to describe the working process of the trained model. For example, for a model with k input variables $x_{i}$, the original model $f\left(x\right)$ can be represented as an explanatory model $h\left(x_{s}\right)$ with reduced input $x_{s}$:

(7) $$f\left(x\right)=h\left(x_{s}\right)=\varphi_{0}+\sum_{i=1}^{k}\varphi_{i}{x^{i}}_{s}$$

where *k* is the number of input features and $\varphi_{0}$ denotes the constant value. When no inputs are used. Inputs x and $x_{s}$ are related by a mapping function. A more vivid explanation is shown in Figure 3, where $\varphi^{0},\;\varphi^{1},\;\varphi^{2},\;\mathrm{and}\;\varphi^{4}$ increases the value of the prediction target and $\varphi^{3}$ decreases the value of the prediction target.

### 3.5. Limitation of the Methodology

While these machine learning models offer several advantages, including high prediction accuracy and the ability to model non-linear relationships, they are not without limitations. One significant limitation is the requirement for a large and diverse dataset to train the models effectively. In situations where data are scarce or not sufficiently varied, the models may overfit the training data and perform poorly on unseen data. Additionally, these models can be sensitive to the quality of the input data; any inaccuracies or inconsistencies in the dataset can lead to biased or incorrect predictions. Another limitation is that while tree-based models can identify relationships between variables, they do not inherently provide a mechanistic understanding of the underlying processes. This means that while they can predict outcomes based on input data, they may not fully capture the underlying physical or chemical phenomena influencing AAM strength development. Furthermore, the models used in this study were optimized for the specific dataset at hand, and their applicability to other types of AAMs or different environmental conditions may be limited [43].

## 4. Results & Discussion

### 4.1. Hyper-Parameter Tuning Results

Setting appropriate hyper-parameters is crucial for developing excellent ML models, and optimized hyper-parameters can maximize the predictive performance of ML algorithms. In this study, the models all underwent hyper-parameter optimization using the BOA algorithm and the 5-fold CV approach.

**Figure 4:**
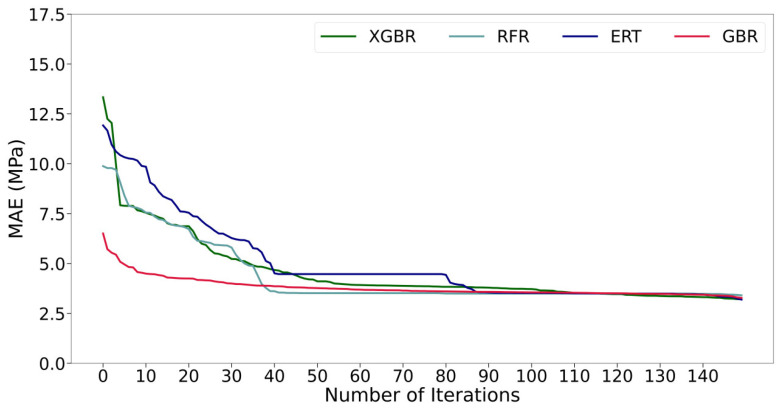
MAE of the ML model as a function of the number of iterations.

Figure 4 depicts the curves illustrating the variation of MAE with the number of iterations for these four ML models. In detail, the hyper-parameters obtained from the 5-fold CV are averaged in each iteration, and this value is used to evaluate the prediction performance of each ML model in subsequent operations. Obviously, all models gradually converge with the increase in the number of iterations, and the prediction performance of all models has been basically exerted when the number of iterations reaches about 110. This suggests that the BOA algorithm excels in fine-tuning the hyper-parameters of tree-based machine learning models. Compared with the other three ML models, GBR converges faster and has a smaller final MAE value overall. The tuned hyper-parameters of each ML model are listed in Table 3.

### 4.2. Prediction Performance of the ML Models

**Figure 5:**
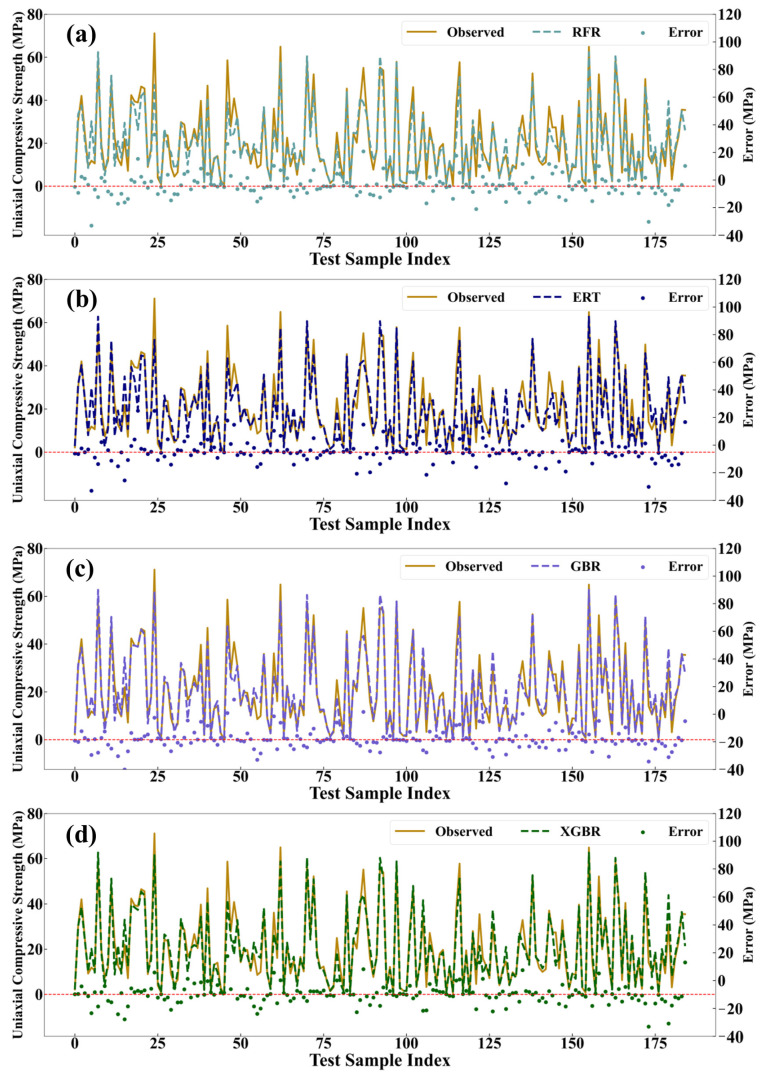
The predicted value and the observed value on the training setwitch (**a**) RFR; (**b**) ERT; (**c**) GBR; (**d**) XGBR. The dashed red line indicates the zero error baseline, where prediction error is zero.

The prediction performances of the ML models with the optimal hyper-parameters on the training set and the test set are compared in this section. In Figure 5, the horizontal axis represents the serial numbers of each data group in the training set, comprising a total of 185 data groups (30% of the entire dataset). The left vertical axis corresponds to the UCS values of the AAMs, with the solid and dashed lines representing the observed and predicted UCS, respectively. The right vertical axis represents the error values between the observed and predicted values. It can be seen that in the four ML models, except for a few exceptions, most of the errors are pretty small, indicating the excellent prediction performance of the four ML models.

**Figure 6:**
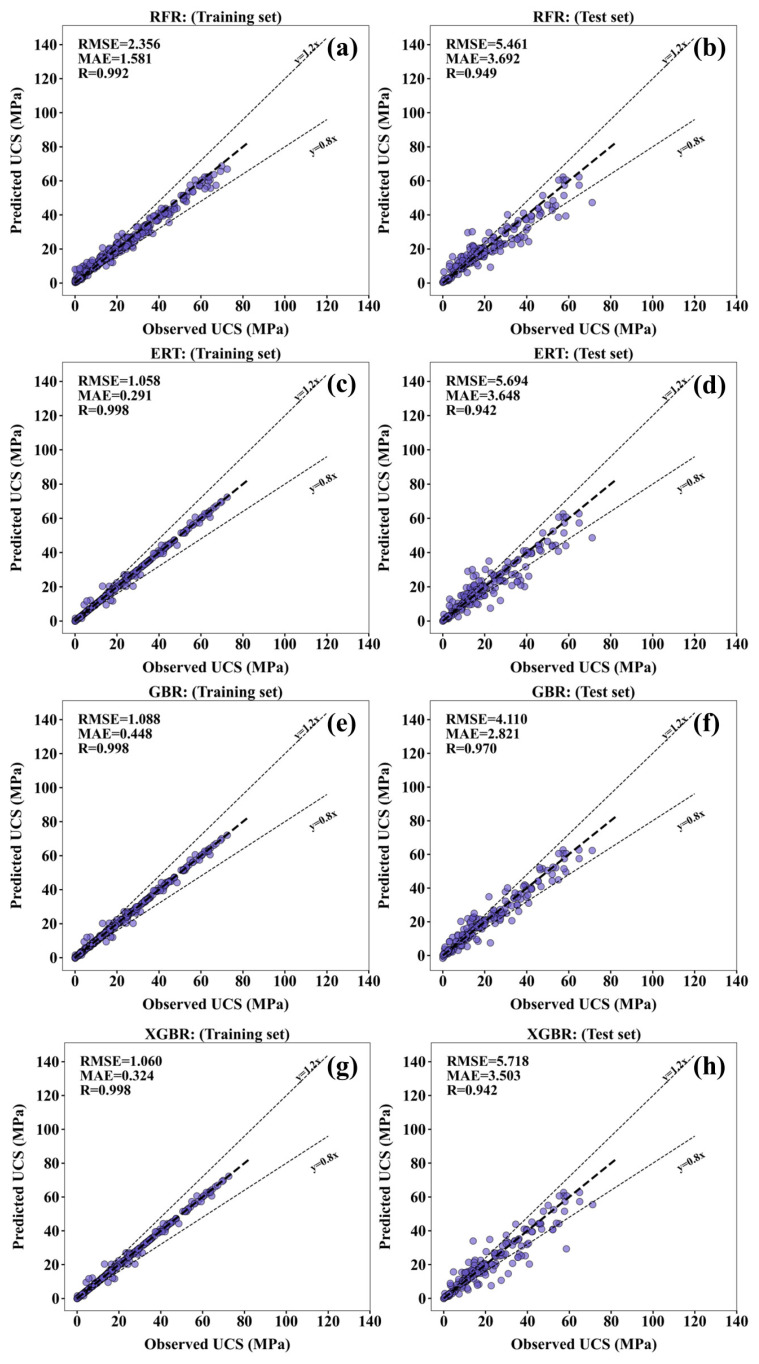
Correlation between the predicted value and the observed value for training and test sets across four machine learning models: (**a**,**b**) RFR, (**c**,**d**) ERT, (**e**,**f**) GBR, and (**g**,**h**) XGBR.

Figure 6 illustrates the correlation between the predicted and observed values of each machine learning model on both the training and test sets. In general, the lower UCS values tend to result in higher prediction errors, whereas higher UCS observation values correlate with improved prediction accuracy. This is because the data points with lower UCS values are not enough for training. Therefore, more mixtures with lower UCS should be collected in the future for training the models. The various performance evaluation indices of the four ML models are shown in Table 4. In the test set, GBR exhibits the highest R value (0.970) among the four ML models. Additionally, the MAE value (2.821 MPa) and RMSE value (4.110 MPa) of GBR are also the lowest.

**Figure 7:**
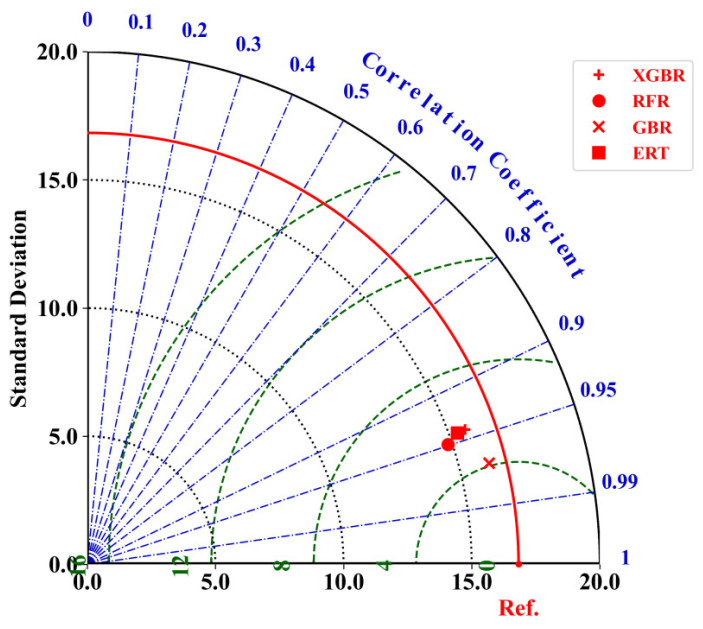
Taylor diagram comparing the prediction performance of four machine learning models (XGBR, RFR, GBR, and ERT) using Pearson correlation coefficient, standard deviation, and RMSE. The red curve represents the reference line (Ref.), and the green dashed lines represent standard deviation contours. The blue dashed lines indicate correlation coefficient contours, with values ranging from 0 to 1.

A Taylor diagram is a common means to compare the performance of various ML models [87] using R, RMSE, and standard deviation (SD) as standards. The prediction performance of each ML model will appear in the Taylor diagram in the form of points, and the closer a point is to Ref., the higher the R value of the ML model represented by the point, and the lower the RMSE and SD values, the better the prediction performance. As shown in Figure 7, among the four ML models of RFR, ERT, GBR, and XGBR, the point representing GBR is closest to Ref., which means that compared with other models, GBR has the most outstanding prediction performance.

### 4.3. Feature Analysis of the Input Variables

After adjusting the hyper-parameters by the BOA algorithm, the GBR is better than other models in terms of prediction accuracy, and then we use the trained GBR to explain the importance of each input variable to the UCS of AAMs and the influence mechanism.

**Figure 8:**
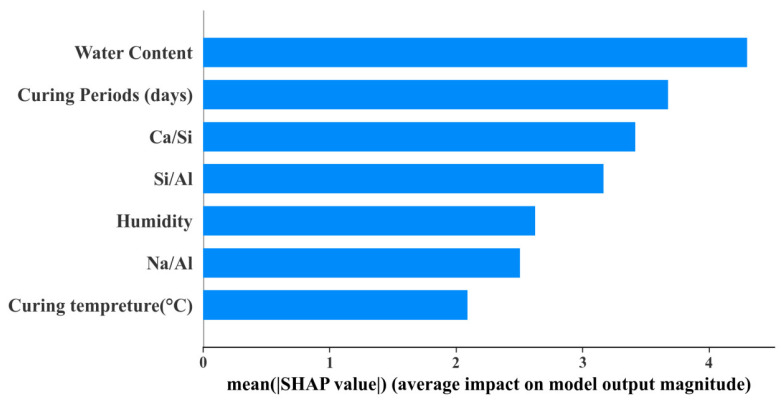
Importance index of input variables.

In general, the degree to which GBR’s prediction accuracy decreases can be observed by excluding an input variable in the dataset, and the importance of that variable among all input variables can be inferred [88,89]. As shown in Figure 8, among all the input variables, the importance of water content is much higher than that of other variables, and its importance index exceeds 4. The variable curing periods rank second and are followed by the Ca/Si ratio and Si/Al ratio, with the SHAP value between 3~3.5. It can be also seen that the importance coefficient of curing temperature is the lowest (2.1). This indicates that the strength of FA-GGBS-based AAMs can be well developed at ambient temperature. In the next section, we will focus on the mechanism of the most important variable water content on UCS, and the internal factors, such as the Ca/Si ratio, Na/Al ratio, and Si/Al ratio, also deserve attention [90].

**Figure 9:**
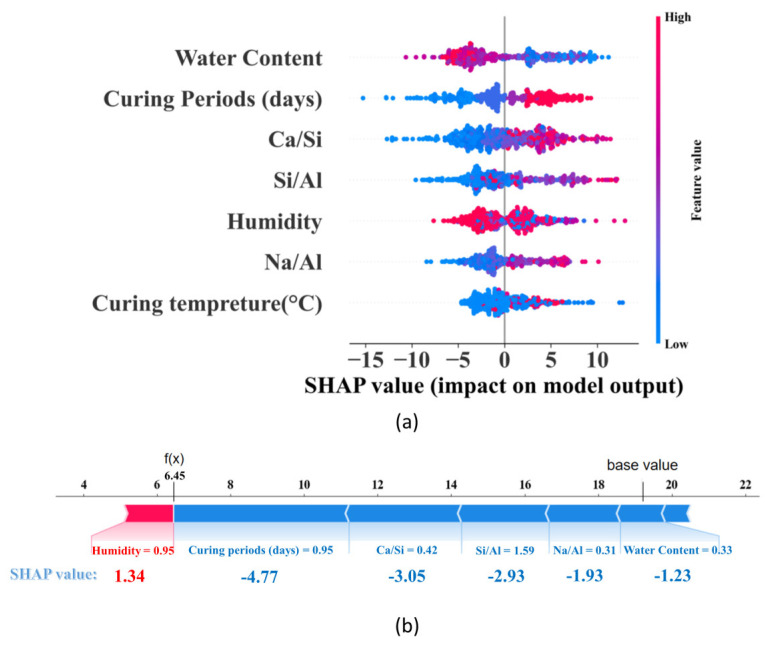
SHAP analysis. (**a**) SHAP violin diagram; (**b**) Local interpretation of SHAP in the first set of data.

The SHAP value was used to analyze the impact of all input features on the UCS development of AAMs [85]. Observing from Figure 9a, it can be seen that for the “water content” the red dot on the far left shows that when the input value of water content is high, it has a negative impact on the UCS development of AAMs, and the strength of AAMs is reduced by about 10 MPa at most. Reduction of water content can increase the strength by 5–10 MPa.

Figure 9b shows a local interpretation of the strength of the AAMs for the first sample in the data set. In the figure, the predicted UCS value for the first sample is 6.45 MPa, and the input values of humidity, curing periods, Ca/Si ratio, Na/Al ratio, Si/Al ratio, and water content are 95%, 3 days, 0.42, 1.59, 0.31, and 33%, and their SHAP characteristic values are 1.34, −4.77, −3.05, −2.93, −1.93, and −1.23, respectively. This indicates that the humidity positively influences the strength of the material and the other factors have a negative effect, which is consistent with the SHAP violin plot shown in Figure 9a.

### 4.4. Sensitivity Study

The decrease in water content does not increase the strength of the material continuously. When its value exceeds a certain threshold, the positive effect on the material’s strength development begins to decrease, and negative impacts may even occur. In fact, factors such as the Ca/Si ratio, curing temperature, and other influencing variables can exhibit a “rebound” phenomenon. Because of this, finding the optimal input values for each variable and explaining the reasons for the occurrence of such phenomena has become the key to exploring the mechanisms through which various input variables affect the strength of AAMs.

**Figure 10:**
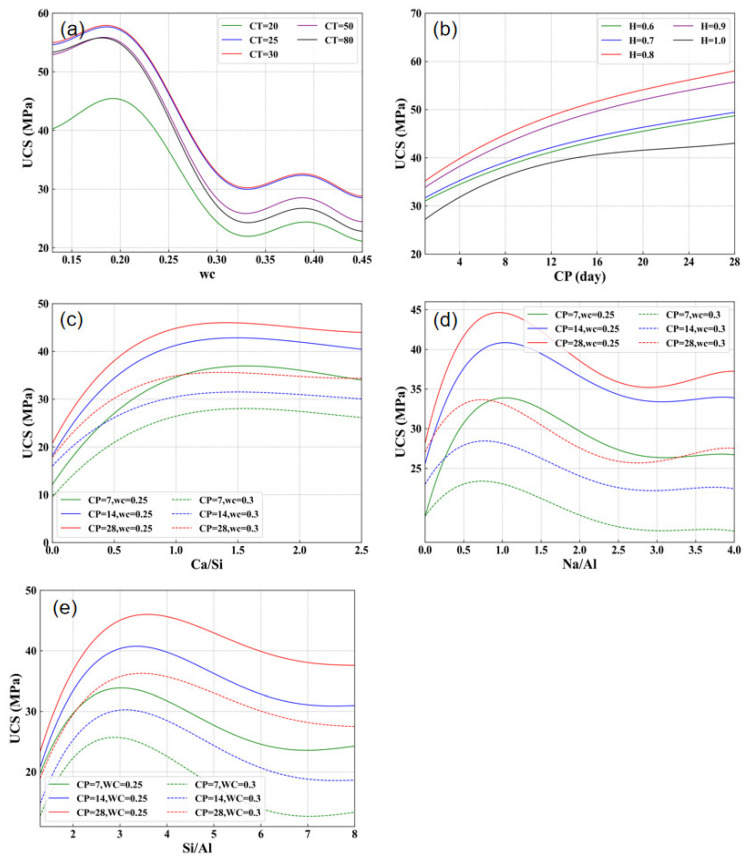
Variation curves of UCS for FA-GBFS-based AAMs generated by the GBR model. The curves illustrate the interdependency of variables: (**a**) Water Content (WC) at different curing temperatures (CT), (**b**) UCS variation with curing periods (CP) at varying levels of ambient humidity (H), (**c**) UCS as a function of the Ca/Si ratio, (**d**) UCS variation with Na/Al ratio under different water content conditions, and (**e**) UCS as a function of the Si/Al ratio. Each curve represents a specific FA-GBFS AAM mixture as modeled by the GBR, highlighting the interactions between these critical input variables.

Figure 10 shows the change of the predicted UCS with changing the influencing variables. It can be seen from Figure 10a that for each temperature, the UCS increases with increasing water content from 0.12 to 0.2. However, beyond 0.2, the UCS decreases and ultimately enters a relatively stable fluctuation state after 0.3. This phenomenon aligns with some previous research findings [91,92,93,94]. This occurs because alkali activation primarily involves chemical reactions between dissolved silicate and aluminate ions, and water acts as a transport fluid during the alkali activation of materials. This not only improves the degree of complete reaction of these salt ions but also promotes the formation of the gel phase [25,91]. However, too high water content can also lead to an increase in the porosity of AAMs, which in turn leads to a rapid decrease in material strength [92,93,95]. An obvious increase is observed with increasing temperature from 20 °C to 30 °C because the activation temperature of fly ash generally requires temperatures around 30 °C to 85 °C. Before reaching 30 °C, the internal fly ash of AAMs is not fully activated, and the strength development varies significantly with temperature changes. After 30 °C, the internal fly ash of AAMs is essentially involved in the reaction, and the strength development stabilizes [61].

Figure 10b illustrates a steady increase in the strength of AAMs with curing time. The strength of AAMs typically increases rapidly in the first few days to weeks, but the reaction continues at a slower rate for an extended period. This prolonged reaction contributes to further densification and strengthening of the material, leading to continued strength development beyond the initial curing period [96,97,98]. The increase in external humidity from 0.6 to 0.8 is beneficial to the development of UCS in AAMs. However, excessive curing humidity can negatively impact the strength development of AAMs. Firstly, high humidity conditions can lead to increased water content in the AAMs, potentially diluting the concentration of alkaline activators. Also, high humidity may result in the leaching of alkaline components from the AAMs. This leaching process can weaken the structure by removing essential components needed for the chemical reactions that contribute to strength development [99,100,101,102].

As shown in Figure 10c, the strength of the geopolymer continues to increase with increasing the Ca/Si ratio to 1.3 [103]. The calcium ions in AAMs primarily originate from GBFS. Calcium ions form strong cohesive planes with the negatively charged layers of C-(N-)A-S-H gel, accelerating the hardening process of AAMs. This promotes the formation and precipitation of nuclei as C-(N-)A-S-H gel, ultimately leading to the rapid formation of AAMs gel. These gels gradually encapsulate the fly ash particles in subsequent reactions, forming a complete matrix [103,104,105]. Meanwhile, when the Ca/Si is too high in the C-(N-)A-S-H gel, the average chain length of the aluminosilicate chains (Q²) is shorter, and there are fewer cross-linking groups (Q³). This leads to a decrease in the strength of AAMs [106].

Figure 10d demonstrates that when the Na/Al ratio approaches 1, the maximum UCS for AAMs is achieved. The sodium element in FA-GBFS AAMs primarily originates from the alkaline activator; an appropriate amount of alkaline activator maximally catalyzes the reaction between slag and fly ash [107]. It is known that $M_{n}{\left[-{\left(SiO_{2}\right)}_{z}-AlO_{2}\right]}_{n}\cdot wH_{2}O$ is the empirical formula of AAMs, where M is the alkali metal elements such as Na^+^, K^+^, etc.; z is the Si/Al ratio, n is the degree of polymerization, and w is the number of bound water [108,109]. This indicates that a Na/Al of 1 can lead to optimal polymerization, resulting in a more cross-linked and denser gel structure. This denser structure enhances the mechanical properties of AAMs. Figure 10d also indicates that the water content has some influence on the optimal value of the Na/Al. When the water content is 0.25, the optimal value of the Na/Al ratio is closer to 1. However, when the water content increases to 0.3, the optimal value of the Na/Al ratio decreases (to around 0.8). This is because when the surrounding water content increases, more aluminum ions transform into crystalline aluminum and are preserved, leading to a decrease in the optimal value of the Na/Al ratio [110,111].

Figure 10e demonstrates that the strength of AAMs increases with increasing the Si/Al ratio until 3. The products of AAMs can be roughly categorized into three types, as delineated by Davidovits et al. [112,113,114]: -Si-O-Al-(PS) type, -Si-O-Al-O-Si-(PSS), and -Si-O-Al-O-Si-O-Si-(PSDS). When the Si/Al ratio increases, the number of -Si-O-Si- chemical bonds in the products of AAMs increases, while the number of -Si-O-Al- chemical bonds decreases. In comparison to -Si-O-Al- bonds, -Si-O-Si- bonds exhibit higher strength and are more challenging to form [115,116]. Exactly because of this, to generate more PSS-type and PSDS-type chemical bonds with higher strength, it is necessary to increase the Si/Al ratio higher than the theoretical value (2~3). This also explains why the actual peak strength of AAMs consistently occurs around a Si/Al ratio of 3 or higher [117].

## 5. Validation of the Design Model by Laboratory Experiments

The trained ML model is used to help design high-performance binary AAMs. A few mixtures of AAMs are designed and cast in the laboratory to verify the proposed method in this section.

### 5.1. Raw Materials

The precursor for the synthesis of AAMs consists of FA and GBFS, provided by Jintaicheng Company, Liaoning, China. The chemical composition of the precursor was determined using X-ray fluorescence (Rigaku ZSX Primus 2, Rigaku Corporation, Tokyo, Japan), as shown in Table 5. The particle size distribution of the precursor was measured by laser diffraction (Malvern Mastersizer 2000, Malvern Panalytical, Malvern, UK), and their particle distributions are listed in Table 6. The particle size distribution of FA showed that the d10, d50, and d90 values were 4.37 µm, 60.03 µm, and 176.81 µm, respectively. This indicates that the majority of FA particles are within the fine to medium size range, which is conducive to effective reaction and gel formation when used in AAMs. The particle size distribution for GBFS showed d10, d50, and d90 values of 1.07 µm, 8.35 µm, and 30.56 µm, respectively. The finer particle size of GBFS, compared to FA, enhances its reactivity, contributing to the formation of a dense and durable matrix in the resulting AAM.

**Figure 11:**
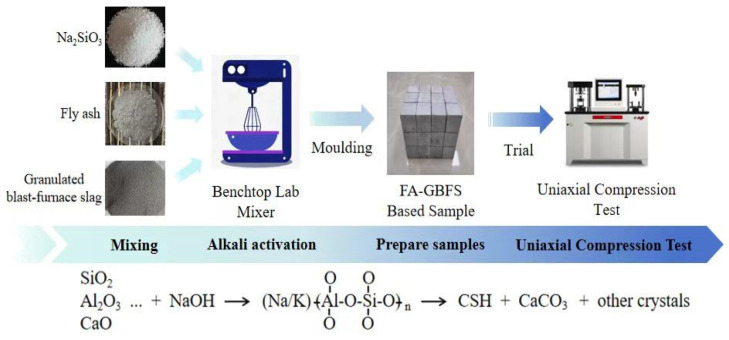
Preparation of binary AAMs with fly ash and slag.

Na_2_SiO_3_ was selected as the alkali activator, purchased from Youso, China, with a modulus of 1.4 (SiO_2_: Na_2_O = 1.4). Water was sourced from laboratory-grade water. The experimental procedure is illustrated in Figure 11.

The high CaO content in GBFS, combined with its appropriate SiO_2_ and Al_2_O_3_ levels, ensures that the precursor material is highly reactive and capable of forming a strong, durable binder when activated. This compliance with the CaO/(SiO_2_ + Al_2_O_3_) > 1.00 criterion underscores the suitability of the materials used in this research for producing high-performance AAMs, further validating the results obtained from the study [24].

### 5.2. Sample Preparation

The mixing proportions of the experimental samples are shown in Table 7, and the elemental ratios are presented in Table 8. The SiO₂/Na₂O molarity ratio in the sodium silicate solution was maintained at 1.4, ensuring sufficient silica availability to react with sodium ions and form a stable, cohesive gel matrix. The sodium silicate was added in a fixed proportion of 8% by weight relative to the total mass of the precursors, a dosage determined through preliminary testing to provide an optimal balance between workability and mechanical performance, effectively enhancing the strength and durability of the AAMs.

The experimental procedure involved using a planetary mixer to blend fly ash, slag, and alkali activator in a 5 L mixing bowl. The dry mixing process lasted for 3 min, followed by a slow, uniform addition of water while continuing to stir for an additional 3 min. The prepared AAMs were then cast into sample molds and subjected to approximately 2 min of vibration on a vibrating table until air bubbles were eliminated. Subsequently, the samples were allowed to cure at room temperature (approximately 25 ℃, with a relative humidity of 40% to 50%) and covered with plastic film to minimize excess water evaporation. After 24 h, demolding was carried out, and the specimens were placed in sealed plastic bags. They were then cured for an additional 72 h at room temperature before removing them from the plastic bags for further curing until testing.

### 5.3. Experimental Results

**Figure 12:**
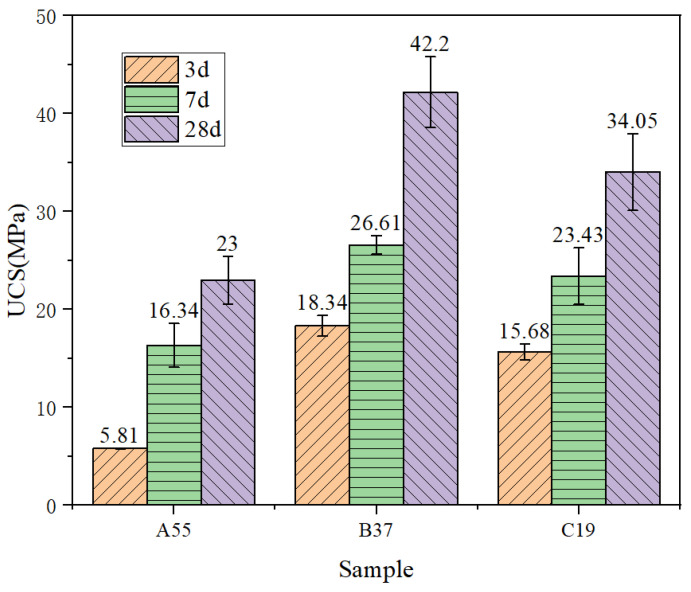
UCS data for each sample.

According to the provisions of GB/T 50081-2002 [3], a universal testing machine with a capacity of 2500 kN (YAWS-2500J, Jinan Yangyi Instrument Co., Ltd., Jinan, China) was used to test specimens of dimensions 50 mm × 50 mm × 50 mm at 3, 7, and 28 days at a loading rate of 0.5 MPa/s. The obtained UCS results are listed in Figure 12.

### 5.4. Simulation Results

**Figure 13:**
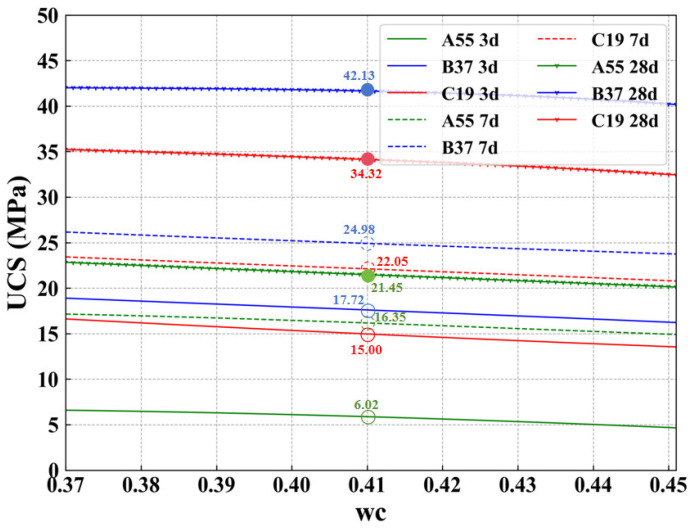
Simulation results.

The UCS values of the mixtures (Table 7) are predicted using the trained ML model (GBR) with a curing temperature of 25 °C and humidity of 41%. The predicted results are depicted in Figure 13. It can be observed that the predicted results are very close to the experimental results. This is also indicated by the minor RMSE and MAE values shown in Table 9. This suggests that the GBR model, optimized through the BOA algorithm, exhibits excellent predictive performance and can be used to design high-performance AAMs. It is worth noting that due to the extensive size of the ML dataset, we could not conduct field tests for every situation. Strictly speaking, the experimental results in this chapter specifically support studies conducted at “41% water content,” and variability in other water content conditions cannot be ruled out. This work still requires further collaboration from researchers to continually refine and improve the conclusions.

## 6. Conclusions

This study first establishes four tree-based ML models to explore the development mechanism and influencing factors of the UCS of AAMs. Utilizing the BOA algorithm for hyper-parameter optimization, 616 sets of data gathered from published literature are utilized for training and testing the ML models. Finally, the following conclusions were reached through the machine learning modeling, with effective results specifically obtained on the tested samples at a water content of 41%, which may not directly sustain all the presented conclusions:

(1) The performance of GBR is the most outstanding, with an R value of 0.970, an MAE of 2.821 MPa, and an RMSE of 4.110 MPa. The GBR model is recommended for molding the UCS of binary AAMs.
(2) The water content and curing periods were the most important variables affecting the development of UCS of AAMs, while humidity had a minimal effect on the UCS of AAMs.
(3) To design high-performance AAMs, it is recommended to maintain the Ca/Si, Na/Al, and Si/Al ratios at approximately 1.3, 1, and 3, respectively. Additionally, the moisture content should be around 0.2 while maintaining the temperature and humidity at approximately 30 °C and around 0.8, respectively.

Future work should focus on expanding the dataset to include a broader range of precursor compositions and environmental conditions to enhance the generalizability of the machine learning models. Investigating the long-term durability of AAMs under various environmental conditions, including exposure to aggressive chemicals and freeze-thaw cycles, is also essential. Furthermore, exploring the effects of different alkaline activators and their combinations could lead to more cost-effective and environmentally friendly AAM formulations. Finally, pilot-scale field studies are needed to validate the laboratory findings and assess the practical applicability of the optimized AAM formulations in real-world construction projects.

## Figures and Tables

**Table 1:** Overview of dataset statistics.

Type	Variables	Min	25%	50%	75%	Max	Mean	SD
Input	Si/Al	1.37	1.70	2.07	2.76	9.61	2.46	1.16
	Na/Al	0.00	0.31	0.53	0.90	4.46	0.69	0.58
	Ca/Si	0.05	0.34	0.66	0.98	3.10	0.77	0.60
	Water Content	0.01	0.16	0.26	0.34	0.50	0.27	1.34
	Curing Temperature (°C)	20.00	20.00	21.00	25.00	80.00	28.67	18.39
	Humidity	0.50	0.75	0.95	0.98	1.00	0.86	0.18
	Curing Periods (days)	1.0	3.00	7.00	28.00	28.00	14.06	11.03
Output	UCS (MPa)	0.00	5.19	14.93	27.95	72.46	19.17	16.90

**Table 2:** The range of hyper-parameter tuning for each model.

Model	Max_Depth		Max_Features		Min_Samples_Leaf		Min_Samples_Split		N_Estimators		Learning_Rate	
	Min	Max	Min	Max	Min	Max	Min	Max	Min	Max	Min	Max
RFR	1	30	1	7	1	40	2	40	100	500		
ERT	1	30	1	7	1	40	2	40	50	500		
GBR	1	30	1	7	1	30	2	30	200	800	0	1
XGBR	1	30	1	7	1	40	2	40	50	500	0	1

Note: Max_Depth: Maximum Depth (of the decision trees in the model); Max_Features: Maximum Features (the number of features to consider when looking for the best split); Min_Samples_Leaf: Minimum Samples per Leaf (the minimum number of samples required to be at a leaf node); Min_Samples_Split: Minimum Samples per Split (the minimum number of samples required to split an internal node); N_Estimators: Number of Estimators (the number of trees in the ensemble for methods like Random Forest or Gradient Boosting); Learning_Rate: Learning Rate (a parameter that controls the contribution of each tree in boosting algorithms).

**Table 3:** List of hyper-parameters used in ML models.

Model	Max_Depth	Max_Features	Min_Samples_Leaf	Min_Samples_Split	N_Estimators	Learning_Rate
RFR	24	4	1	2	412	
ERT	16	7	1	2	50	
GBR	4	3	1	2	800	0.077
XGBR	4	3	40	40	282	0.126

**Table 4:** The values of various performance evaluation indicators of the four ML models.

	RFR		ERT		GBR		XGBR	
	Train Set	Test Set	Train Set	Test Set	Train Set	Test Set	Train Set	Test Set
R	0.992	0.949	0.998	0.942	0.998	0.970	0.998	0.942
R^2^	0.981	0.895	0.996	0.886	0.996	0.940	0.996	0.885
MAE (MPa)	1.581	3.692	0.291	3.648	0.448	2.821	0.324	3.503
RMSE (MPa)	2.356	5.461	1.058	5.694	1.088	4.110	1.060	5.718

**Table 5:** Oxide composition of FA and GBFS (%).

Oxide	SiO_2_	Al_2_O_3_	Fe_2_O_3_	CaO	MgO	SO_3_	Other Minor Oxides
FA	52.1	34.3	5.42	2.77	0.49	1.45	3.47
GBFS	28.04	14.33	0.88	44.99	5.086	3.15	3.524

**Table 6:** Particle size analysis of solid precursors.

Solid Precursors	$d_{10}\;\left(\mu m\right)$	$d_{50}\;\left(\mu m\right)$	$d_{90}\;\left(\mu m\right)$
FA	4.37	60.03	176.81
GBFS	1.07	8.35	30.56

**Table 7:** Mix proportions of the AAMs (sodium silicate is in the form of powder).

Sample ID	Components (wt.%)			
	FA	GBFS	Water	Sodium Silicate
A55	50	50	41	8
B37	30	70	41	8
C19	10	90	41	8

**Table 8:** Elemental ratios of mixed AAMs.

Sample ID	Ca/Si	Si/Al	Na/Al
A55	0.573	1.562	0.230
B37	0.869	1.667	0.275
C19	1.246	1.824	0.342

**Table 9:** Error analysis.

Sample ID	MAE (MPa)	RMSE (MPa)
A55	0.59	0.52
B37	0.77	0.58
C19	0.78	0.52

## Data Availability

Data are contained within the article and Supplementary Materials.

## Supplementary Materials

The following supporting information can be downloaded at: https://www.mdpi.com/article/10.3390/ma17184573/s1.

## Abbreviations and Their Full Names in the Paper

AAMs	Alkali-Activated Materials
R	Correlation Coefficient
UCS	Uniaxial Compressive Strength
FA	Fly Ash
R²	Coefficient Of Determination
RFR	Random Forest Regression
GBFS	Granulated Blast Furnace Slag
MAE	Mean Absolute Error
ERT	Extremely Randomized Trees
OPC	Ordinary Portland Cement
RMSE	Root Mean Square Error
GBR	Gradient Boosting Regression
ML	Machine Learning
SD	Standard Deviation
XGBR	Extreme Gradient Boosting Regression
SVM	Support Vector Machine
SHAP	SHapley Additive exPlanations
CART	Classification And Regression Trees
RF	Random Forest
WC	Water Content
PS	-Si-O-Al-
ANN	Artificial Neural Network
CP	Curing Periods
PSS	-Si-O-Al-O-Si-
LSTM	Long Short-Term Memory
CT	Curing Temperature
PSDS	-Si-O-Al-O-Si-O-Si-
BOA	Bayesian Optimization Algorithm
H	Humidity
